# Are There Ovarian Responsive Indexes That Predict Cumulative Live Birth Rates in Women over 39 Years?

**DOI:** 10.3390/jcm11082099

**Published:** 2022-04-08

**Authors:** Sara Cesarano, Paul Pirtea, Achraf Benammar, Dominique De Ziegler, Marine Poulain, Alberto Revelli, Chiara Benedetto, Alexandre Vallée, Jean Marc Ayoubi

**Affiliations:** 1 Hospital FOCH, 92150 Suresnes, France; sara.cesarano2@gmail.com (S.C.); a.benammar@hopital-foch.com (A.B.); dom@deziegler.com (D.D.Z.); marine.poulain@hopital-foch.com (M.P.); al.vallee@hopital-foch.com (A.V.); jm.ayoubi@hopital-foch.com (J.M.A.); 2 University of Torino, 10124 Torino, Italy; aerre99@yahoo.com (A.R.); chiara.benedetto@unito.it (C.B.); 3 Department of Epidemiology-Data-Biostatistics, Delegation of Clinical Research and Innovation, Foch Hospital, 92150 Suresnes, France

**Keywords:** ovarian response indexes, FORT, FOI, OSI, controlled ovarian stimulation, cumulative outcomes, ART

## Abstract

*Objective:* Ovarian response indexes have been proposed in assisted reproductive technology (ART) in order to optimize live birth rates (LBR), adjusting ovarian stimulation (OS), and minimizing risks. Gonadotropin doses are commonly adjusted according to ovarian reserve parameters, including antral follicle count (AFC), anti-Mullerian hormone (AMH), and basal follicle stimulating hormone (FSH) levels. The retrospective assessment of ovarian responses allows one to identify three primary indexes: (i) follicular output rate (FORT), the ratio of the number of pre-ovulatory follicles obtained at OS completion over AFC; (ii) follicle oocyte index (FOI), the ratio of oocytes retrieved over AFC; (iii) ovarian sensitivity index (OSI), the ratio of oocytes retrieved over the total gonadotropin dose administered. In recent publications, these indexes were reported to predict ART outcome. In the present study, we assessed the ability of these indexes to predict cumulative ART outcome in women ≥39 years. *Materials and Methods:* Retrospective cohort study. All patients ≥39 years who performed their first ART cycle with an antagonist protocol in our center between 01/2018 and 04/2020 were included. Patients with basal FSH > 20 IU/l, AMH < 0.1 ng/mL and severe male factors (azoospermia with testicular biopsy) were excluded. All patients received both recombinant FSH and human menopausal gonadotropin (hMG). Cumulative live birth rate (cLBR) was the primary outcome. Secondary outcomes included: the number of MII oocytes, cumulative implantation (cIR), and usable blastulation rates. Logistic regressions were performed to assess the predictive values of FORT, FOI, and OSI in cLBR and embryo culture success. For each parameter, the ability of the logistic regression models to predict embryo culture success was quantified by the area under the ROC curve (AUC). Only the significant findings related to FORT, FOI, and OSI were included in the multiple logistic regression model. Linear regression models were performed between cIR, cLB, FORT, FOI, and OSI. Each statistic model was adjusted for age. Concerning OR for OSI, values were multiplied *100 due to the very low value. *Results:* 429 patients met the inclusion criteria. There were 298 obtained usable blastocysts after ART treatment. Age-adjusted OSI was significantly associated with cLBR [OR = 17.58 95% CI (5.48–56.40), AUC = 0.707 95% CI (0.651–0.758)) and cIR (beta = 30.22 (SE: 7.88), *p* < 0.001, R2= 0.060). Both FOI (OR = 6.33 95% CI (3.27–12.25), AUC = 0.725 95% CI (0.675–0.771), R2 = 0.090, *p* < 0.001) and OSI (OSI*100; OR = 1808.93 95% CI (159.24–19,335.13), AUC = 0.790 95% CI (0.747–0.833), R2 = 0.156, *p* < 0.001) were independently, when age adjusted, associated with embryo culture success. OSI showed a main performance to explain successful embryo culture than FOI (R2 = 0.156 vs. R2 = 0.090, *p* < 0.001). In the age-adjusted linear regression model, FOI (R2 = 0.159, *p* < 0.001), OSI (R2 = 0.606, *p* < 0.001), and FORT (r2 = 0.030, *p* < 0.001) were predictive of the number of MII oocytes collected. Furthermore, for OSI (r2 = 0.759, *p* < 0.001) and FOI (r2 = 0.297, *p* < 0.001), the correlation with the number of metaphase II oocytes collected was significantly higher in the non-linear regression model. *Conclusions*: Our findings suggest that the best index, among those analyzed, to predict cIR and cLBR, is OSI. Both OSI and FOI predict embryo culture with success, but OSI is more accurate. OSI, FOI, and FORT are significantly related to the number of MII oocytes obtained.

## 1. Introduction

The ovarian response to stimulation is one of the most studied parameters in assisted reproductive technologies (ART), in order to optimize outcomes while minimizing risks. Indeed, the magnitude of the response to ovarian stimulation (OS) has a direct impact on the number of oocytes harvested, which is one of the primary factors affecting the ART yield, and in turn pregnancy rates [1].

Classically, the number of oocytes retrieved is taken as the main marker of ovarian responsiveness to gonadotropin (Gn) stimulation. When looking at fresh embryo transfers, the retrieval of 15–18 oocytes was found to be associated with optimal IVF outcome. Secondary outcome measures include the total dose of gonadotrophins administered, duration of stimulation, and peak serum E2 levels [2].

To tailor and optimize OS outcome, gonadotrophins doses are adjusted according to ovarian reserve parameters, including antral follicle count (AFC), anti-Müllerian hormone (AMH), and basal follicle stimulating hormone (FSH) levels [3].

While AMH and AFC provide a good estimate of the number of oocytes harvested, their prediction of live birth rates is limited [4]. These biomarkers represent a “static” snapshot of the individual ovarian reserve; they do not reflect the “dynamic” nature of follicular growth in response to exogenous COS (controlled ovarian stimulation). There is a strong individual variability in the response to stimulation, linked to both extrinsic (gonadotropin dose) and intrinsic factors (FSH receptor polymorphisms) [5] and the individual rhythm of follicular maturation waves [6]. The latter can lead to an unexpected ovarian response to OS. The observation that the total number of oocytes retrieved does not always accurately reflect the ovarian potential has sparked research of other markers of ovarian response. With this in mind, the attention has been focused on qualitative markers of ovarian response. These include follicular output rate (FORT), follicle-to-oocyte index (FOI), and ovarian sensitivity index (OSI), which may better reflect the dynamic nature of follicular growth in response to exogenous gonadotrophins [7].

FORT, defined as the ratio of the number of pre-ovulatory follicles obtained after OS completion over the pool of AFC, was introduced to quantify the ovary’s follicular competence. Introduced by Gallot et al., to make easier the interpretation of relationship between follicle responsiveness to COS and IVF outcome, FORT groups were categorized as low, medium, and high. The three FORT groups were arbitrarily chosen according to whether FORT values were under the 33th percentile (<42%, low FORT group), between the 33th and the 67th percentile (42–58%), or above the 67th percentile (>58%, high FORT group) of distribution. FORT was significantly higher in women who achieved a clinical pregnancy when compared with those who did not (54.4 + 1.3 vs. 47.2 + 1.2%, respectively, *p* < 0.001) [8].

Both the absolute number of oocytes retrieved, and the total gonadotrophin dose are important measures of ovarian responsiveness, therefore, their ratio, OSI, should be a better representation of ovarian responsiveness. It is significantly related to the main biomarkers of the ovarian reserve (AMH and AFC) and it is more closely linked with clinical pregnancy than the number of retrieved oocytes. It has been proposed also to define patients as poor, normal, and high response patterns in COS [9,10].

FOI is the ratio between the total number of oocytes collected at the pick-up, and the number of antral follicles available (AFC). This parameter was proposed as an alternative approach to address the ovarian resistance to gonadotrophins stimulation. Low FOI values imply that only a fraction of available antral follicles was exploited during OS, suggesting that there might be therapeutic opportunities to change the fate of these women in a subsequent OS. Naturally, technical aspects related to oocyte retrieval and triggering final oocyte maturation can influence FOI results.

FORT, FOI, and OSI are considered to be positively related to the outcomes of IVF [8]. Until now, there have been few reports relating these indices to the cumulative ART outcomes, in particular cumulative pregnancy rate. Many of the existing studies in the literature analyses at most only one or two of these and relate these indices only with the number of oocytes recovered. 

This retrospective analysis was carried out with the aim of testing the possible relationship between the indexes FORT, FOI, OSI, and the reproductive outcome as reflected by the cumulative live birth, implantation, and usable blastulation rates.

## 2. Materials and Methods

### 2.1. Subjects

This study retrospectively investigated patients ≥39 years old who underwent their first IVF autologous cycle at Foch’s Assisted Reproductive Technology Center in Suresnes (France) between January 2018 and April 2020. We included all causes of infertility. Exclusion criteria were: basal FSH ≥ 20 IU/l, AMH ≤ 0.1 ng/mL, severe male factors (azoospermia with testicular biopsy), and BMI ≥ 35.

For patients who obtained embryos after the oocyte’s retrieval and IVF, only those who transferred blastocysts were selected. Cumulative live birth rate (cLBR) was the primary outcome. Secondary outcomes included: the number of MII oocytes, cumulative implantation (cIR) and usable blastulation rates.

### 2.2. OS and Embryo Transfer Protocol

All patients underwent GnRH-antagonist protocol and received both recombinant FSH and human menopausal gonadotropin (hMG), which is routinely given in a three/one ratio. The daily dose ranged between 225 and 600 IU, which was individually adjusted according to age, basal FSH, AMH, and AFC. The GnRH antagonist was always administered from the sixth day of OS. Ovarian response was regularly monitored by transvaginal ultrasound (US) examination and serum estradiol measurement. Ovulation was triggered as soon as three or more pre-ovulatory follicles (≥18 mm in diameter) were observed and E2 levels were >1000 pg/mL. The triggering used a combination of human chorionic gonadotropin (HCG) and GnRH agonists or only with GnRH agonists, according to patient’s hyperstimulation risk. Received GnRH trigger, patients with E2 level higher than 3000 and >20 follicles on the trigger day. Oocytes were retrieved 35 h after triggering by transvaginal ultrasound-guided aspiration. Fertilization was achieved either by conventional IVF or intracytoplasmic sperm injection (ICSI), depending on semen parameters. The uterine cavity was routinely evaluated by hysteroscopy to exclude uterine pathologies that might compromise pregnancy potential.

For fresh embryo transfer, patients began the luteal phase support treatment: 100 mg/day of acetyl salicylic acid, 200 mg twice daily of cefixime for three days, from the evening of the egg retrieval, and vaginal progesterone 200 mg twice daily, subcutaneous progesterone 25 mg/day, oral estradiol two mg BID, from the day after the pick-up. For the frozen embryo transfer, the patients started taking oral estradiol at a dose of 2 mg twice daily and acetyl salicylic acid 100 mg/day from the first day of last period. Ultrasound examination of the endometrium was performed between the eighth and the tenth day of the cycle; if the endometrium was seven millimetres or thicker, the patient started taking vaginal and subcutaneous progesterone. The embryo transfer was carried out after five days of treatment. The luteal phase support treatment had to continue until the first pregnancy test (serum hCG assay), which was performed ten days after the embryo transfer, and up to twelve weeks of amenorrhea, in case of pregnancy. Clinical pregnancy was defined as the presence of a gestational sac observed at US scan at around seven weeks of amenorrhea.

### 2.3. FORT, FOI and OSI Calculation

All patients before starting ovarian stimulation underwent a vaginal ultrasound to determine the AFC—the number of all follicles measuring between three and eight millimetres in diameter. FORT was calculated by dividing the number of pre-ovulatory follicles (POF) obtained at ovarian stimulation over the AFC. Pre-ovulatory follicles count was valued on the last vaginal ultrasound check before the trigger. We considered the follicles with medium diameter of 17 mm or more as preovulatory.

FOI was calculated as the ratio between the total number of oocytes picked up at the end of OS and the number of antral follicles available at the start of stimulation (AFC).

OSI was calculated as the number of oocytes retrieved divided by the total administered Gn dose. Concerning OR for OSI, the value was multiplied *100 due to the very low value.

### 2.4. Statistical Analysis

Continuous variables are presented as median + (25th percentile–75th percentile) and were compared using the Mann-Whitney test according to the distribution of the continuous variables. Categorical variables are presented as n (percentage) and were compared using the Chi-squared test or Fisher’s exact test.

Age-adjusted logistic regression models were performed between successful embryo cultures with FORT, FOI, and OSI. A multivariable logistic regression model was performed between successful embryo cultures and significant variables (*p* < 0.05) among FORT, FOI, and OSI. The ability of the logistic regression models (with odds ratio (OR) and 95% CI (confidence interval)) to predict successful embryo culture were quantified by the area under the ROC curve (AUC) with 95% confidence interval (CI), the determination of the R^2^ (adjusted coefficient of determination), the calculation of sensitivity (Se) and specificity (Sp), positive predictive value (PPV), negative predictive value (NPV), and accuracy. Se, Sp, PPV, and NPV were calculated based on performing a confusion matrix of the different models. Linear and age-adjusted linear regression models (with beta: regression coefficient with SE (standard error)) were performed to investigate the relationship between cIR, cLB, the number metaphase II oocytes collected, and the fertilization rate with FORT, FOI, and OSI. Non-linear single-variable regression models were performed to investigate higher correlation between FORT, FOI, and OSI and the different outcomes. Results reported for each age-adjusted model were the adjusted coefficient of determination, R^2^, and the squared partial correlation coefficient, r2, which were used to describe the contribution to FORT, FOI, and OSI for each parameter. Non-linear regression models were performed manually according to the aspects of the distribution of each variable and to present the highest correlation with studied parameters and restraining on one degree polynomial equations with X transformation, including natural logarithm, exponential, square, square root or reciprocal, and no Y transformation. Differences in correlation between models were assessed using Steiger’s Z tests. AUC of ROC curves were compared by the Delong test (Delong E et al. 1988 Comparing the Areas under Two or more correlated Receiver Operating characteristic curves: a nonparametric approach. Biometrics 44:837-845). Statistics were performed using SAS software (version 9.4; SAS Institute, Carry, NC, USA). A *p* value < 0.05 was considered to indicate statistical significance. The data presented here only involved a retrospective review of the centre’s anonymized electronic research database, also used to report the centre’s annual IVF outcomes to national registries. 

The study was authorized by the local ethical committee Foch IRB: IRB00012437.

## 3. Results

In all, 429 patients satisfied the inclusion criteria. Age-adjusted OSI was significantly associated to cLBR (OR = 17.58 95%CI (5.48–56.40), AUC = 0.707 95% CI (0.651–0.758)), and cIR (beta = 30.22 (SE: 7.88), *p* < 0.001, R^2^ = 0.060). No significant association was observed between cLRB and age-adjusted FOI (*p* = 0.317) and age-adjusted FORT (*p* = 0.091); the same results were observed with linear models (age-adjusted FOI, *p* = 0.286 and age-adjusted FORT, *p* = 0.529).

A number of 98 cases obtained usable blastocysts after ART treatment. Between the group of patients who obtained a successful embryo culture and those who did not obtain embryos, there was no statistically significant differences with respect to the type of technique used (IVF/ICSI), smoking in both female and male partners, primary or secondary infertility, or BMI. However, there were significant differences for patients’ age (*p* = 0.010), FSH (*p* < 0.001), AMH (*p* < 0.001), AFC (*p* < 0.001), the number of oocytes retrieved (*p* < 0.001), the dose of gonadotropins administered (*p* < 0.001), and treatment’ duration (*p* = 0.018) (Table 1).

**Figure 1:**
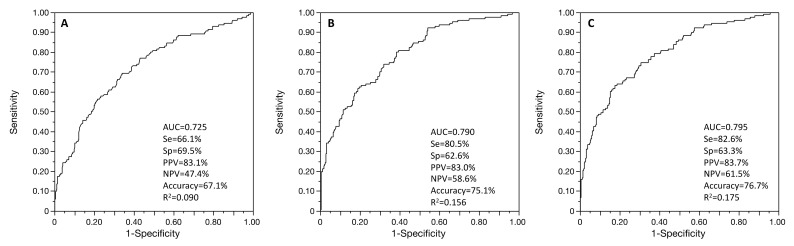
ROC curves of the age-adjusted associations between successful embryo culture and FOI (**A**), OSI (**B**), and multivariable regression with both FOI and OSI (**C**). Se: sensitivity; Sp: specificity; PPV: positive predictive value; NPV: negative predictive value; AUC: area under the ROC curve.

Successful embryo culture was not, when age-adjusted, associated with FORT (*p* = 0.208) but was with FOI (OR = 6.33 95% CI (3.27–12.25), AUC = 0.725 95% CI (0.675–0.771), R^2^ = 0.090, *p* < 0.001) and with OSI (OSI * 100; OR = 1808.93 95% CI (159.24–19,335.13), AUC = 0.790 95% CI (0.747–0.833), R^2^ = 0.156, *p* < 0.001) (Figure 1). When including both OSI and FOI in the age-adjusted model, these two factors remained both independently associated with successful embryo culture (AUC = 0.795 95% CI (0.753–0.834), R^2^ = 0.175) (Figure 1). OSI showed the main performance to explain successful embryo culture, rather than FOI (R^2^ = 0.156 vs. R^2^ = 0.090, *p* < 0.001), but with no added value with the combination of these two parameters when compared to OSI only (R^2^ = 0.175 vs. R^2^ = 0.156, *p* = 0.369). When comparing AUC ROC curves for age-adjusted models, OSI showed a higher performance than FOI (*p* < 0.001), but the combined age-adjusted model OSI + FOI showed no significant difference with age-adjusted OSI only (*p* = 0.581).

**Figure 2:**
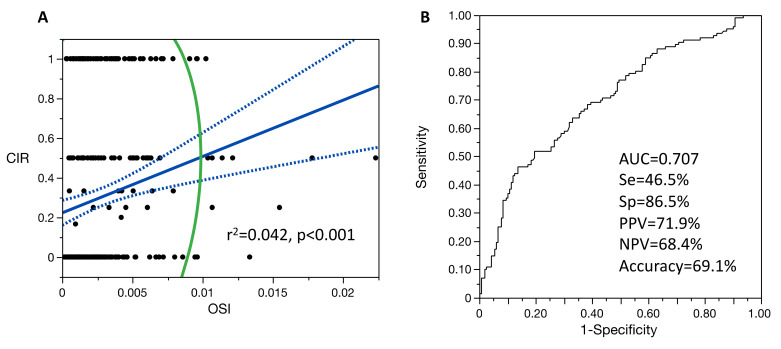
Single-variable linear regression model for the relationship between cIR and OSI (**A**) and ROC curve of the age-adjusted logistic regression model between cIR and OSI (**B**). For figure A: blue line is the fit line of the model with its confidence interval; green line is the 95% density ellipse. Se: sensitivity; Sp: specificity; PPV: positive predictive value; NPV: negative predictive value; AUC: area under the ROC curve.

In the age-adjusted linear regression models, cIR (N = 297 patients) was significantly correlated with OSI (R^2^ = 0.060, *p* < 0.001) (Figure 2), but not FORT (*p* = 0.529) and not FOI (*p* = 0.286). When considering cIR level as binary variable, as cIR > 0 or not, only age-adjusted OSI remained significantly associated (aged-adjusted OR = 17.58 95% CI (5.48–56.41), AUC = 0.706, *p* < 0.001) (Figure 2). No significant differences were observed between non-linear and linear regression models for the relationship between cIR and OSI.

In the age-adjusted linear regression models, cLB (N = 82 patients) was age-adjusted and correlated with FORT (beta = 0.18 (0.09), R^2^ = 0.061, *p* = 0.043), but not with FOI (*p* = 0.316) or OSI (*p* = 0.067).

**Figure 3:**
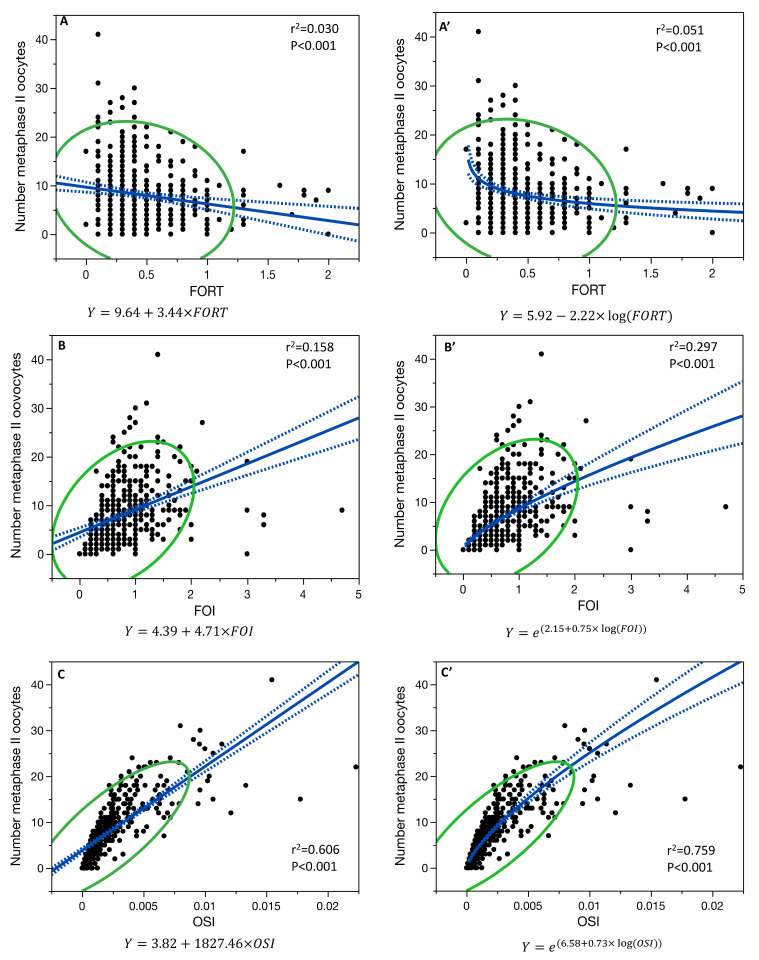
Single-variable linear regression model for the relationship between the number of metaphase II oocytes collected and FORT (**A**), FOI (**B**), and OSI (**C**), and the single-variable non-linear regression model for FORT (**A’**), FOI (**B’**), and OSI (**C’**). Blue line is the fit line of the model with its confidence interval; green line is the 95% density ellipse.

In age-adjusted linear regression models, the number of metaphase II oocytes collected (N = 429 patients) was significantly correlated with FOI (R^2^ = 0.159, *p* < 0.001) and OSI (R^2^ = 0.606, *p* < 0.001) but not with FORT (R^2^ = 0.030, *p* < 0.001). When considering the single-variable linear regression, the number of metaphase II oocytes collected was significantly correlated with FOI (r^2^ = 0.158, *p* < 0.001), but with a significant higher correlation (*p* < 0.001) with the non-linear regression model (r^2^ = 0.297, *p* < 0.001) (Figure 3).

The number of metaphase II oocytes collected and OSI were correlated with a non-linear regression model (r^2^ = 0.759, *p* < 0.001), being significantly higher (*p* < 0.001) than their linear regression (r^2^ = 0.606, *p* < 0.001) (Figure 3).

Moreover, the relationship between the number of metaphase II oocytes collected and FORT was also both significant in the non-linear regression model (r^2^ = 0.051, *p* < 0.001) and in the linear regression model (r^2^ = 0.030, *p* < 0.001) (Figure 3), but with no difference in the two models (*p* = 0.327).

**Figure 4:**
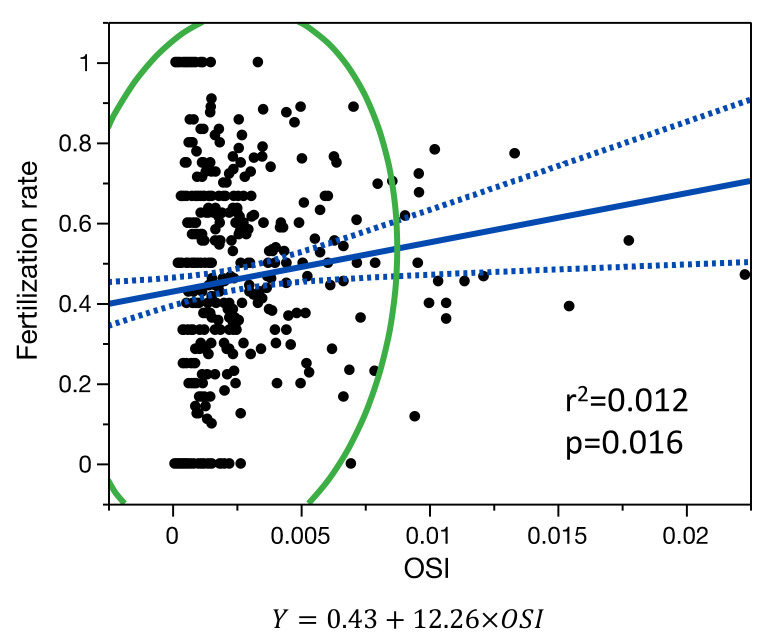
Single-variable linear regression model for the relationship between fertilization rate and OSI. Blue line is the fit line of the model with its confidence interval; green line is the 95% density ellipse.

In the age-adjusted linear regression model, the fertilization rate (N = 426 patients), was only correlated with OSI (R^2^ = 0.027, *p* = 0.013) but not with FORT (*p* = 0.876) and not with FOI (*p* = 0.253) (Figure 4).

No significant differences were observed between non-linear and linear regression models for the relationship between the fertilization rate and OSI.

## 4. Discussion

Our findings suggest that the best index, among those analyzed, that predict cIR and cLBR is OSI. Both, OSI and FOI are significantly related to the number of MII oocytes obtained and predict embryo culture success. Traditionally, IVF success rates have been reported in terms of live birth per fresh cycle or embryo transfer. Sunkara et al. [2] demonstrated that there is an initial increase in fresh LBR with the number of oocytes retrieved; LBR either reaches a plateau or may even decline when more than 15–20 oocytes are harvested. On the other hand, when all fresh and frozen embryos are considered, there is a significant positive association with ovarian response. Cumulative live birth rates (cLBR) are defined as the first live birth following the use of all fresh and frozen embryos derived from a single ovarian stimulation cycle and appear to be a better measurement of IVF treatment success. cLBR increases with the number of oocytes retrieved, suggesting that ovarian stimulation may have a minimal or no detrimental effect on oocyte/embryo quality [11].

This could erroneously suggest that stimulation with higher doses of gonadotropins, and the consequent obtaining of a higher number of oocytes, will give us greater chances of success. However, in the management of our patients, especially in the setting phase of the treatment, we must never forget the two main risks they may face during stimulation: the hyperstimulation risk (OHSS) and the thrombotic risk.

Before starting OS, the assessment of AFC and AMH levels allows prediction of the risk of high ovarian responses, defined as more than 15–20 oocytes retrieved. Gn dose might be planned according to the assessed risk. The use of a GnRH antagonist protocol is beneficial to high-risk women as it markedly decreases the incidence of OHSS without affecting clinical pregnancy ovarian response to COS. Moderate and severe forms of hyperstimulation occur in 3–10% of all IVF cycles; the incidence can reach 20% among high-risk women [12]. The incidence of venous thromboembolic events in women undergoing in vitro fertilization is estimated at being 0.08–0.11% of all treatment cycles, and the incidence of arterial thrombosis is significantly lower [13]. The development of these events is mainly attributed to OHSS. However, the number of retrieved oocytes may be affected by a series of intrinsic factors such as the polymorphic variability of FSH receptors and the individual rhythm and the extent of the follicular maturation waves [14]. When in the final treatment evaluation we only consider the number of oocytes retrieved as an outcome of ovarian response, we do not consider the individual sensitivity of the ovary to the pharmacological stimulus. The number of oocytes retrieved at pick-up does not always represent a real expression of the potential of the ovary to exogenous stimulus. A very low dose of FSH is often used for COS in women with an expectedly high response, which may result in a low oocyte yield and therefore an erroneous classification of the patient as a poor responder. It is therefore essential to evaluate the dose of gonadotropins necessary to develop a certain number of follicles, and to recover as many oocytes as possible. Overwhelming is the evidence of a significant negative effect of smoking on female fertility and also upon clinical outcomes of ART. In particular, there is evidence of decreased clinical pregnancy rate among smokers, in addition to the strong implication of a negative effect on live birth rates, miscarriage rates, ectopic pregnancy rates, and fertilization rates [15]. Different studies have variously reported an increased gonadotropin requirement for ovarian stimulation, lower peak estradiol levels, fewer oocytes retrieved, and higher numbers of canceled cycles [16]. Therefore, smoking could be influencing OSI calculation more than all the other parameters. We evaluated smoking habit in our study population but we did not find statistically significant differences regarding obtaining or not of an embryonic culture.

In the last few years, scientific research in reproductive medicine has focused above all on identifying variables capable of predicting the IVF outcome. The objective is to further individualize ovarian stimulation protocols for optimizing results and reducing costs and complications. Identifying prognostic indices is very complex, as many variables may affect ART success, including the ovarian responsiveness, the number of oocytes available, and their competence.

This is the first study that takes into consideration all three indices, FORT, FOI, and OSI, and correlates them with multiple aspects of ART outcome.

In our study, the FORT index did not prove to be statistically significant in predicting cLBR, cIR, and fertilization rate; it is only statistically significant in predicting the number of metaphase II oocytes collected. This is probably due to index calculation, based on the measurement of the AFC and on the monitoring method used, ultrasound scans during stimulation, which has some limitations:

(a) It is based on the AFC, which is an operator-depending exam
(b) AFC can be affected by the presence of space-occupying structures within the ovary, such as a corpus luteum, a dominant follicle, or, even worse, an ovarian cyst. In these conditions, the measurement is highly inaccurate, and this can lead to a loss of predictive value of the index [17],
(c) oocyte recovery also depends on the choice of trigger timing. This depends on the follicular diameter and above all on the experience/organization of the different ART centres.

The FOI index was statistically correlated only with the number of MII oocytes recovered and the success of embryonic culture. This is probably partially attributable to some of the same limits we talked about regarding the FORT index, as it is also based on the AFC calculation. However, we can affirm that a good correspondence between the follicular count and the number of oocytes retrieved at the pick-up is certainly a sign of a good response to ovarian stimulation.

FOI is considered to be an indirect measure of ovarian response to gonadotropins. A good ovarian response, in quantitative terms (number of oocytes retrieved and percentage of MII oocytes), certainly correlates positively with the achievement of an evolutive embryonic culture.

In our study, OSI is the best index, which is statistically predictive, for all the outcome parameters analyzed: cLBR, cIR, success of embryo culture, and number of MII oocytes.

The OSI links the number of retrieved oocytes to the degree of hormonal stimulation, expressing how many units of exogenous gonadotropins are needed to obtain each oocyte [16]. This suggests that the patients with a poorly responsive ovary, who need a high gonadotropin dose, are ab initio less prone to pregnancy, as they may be pharmacologically forced to produce more oocytes, but those of poor quality. This emphasizes the independent information given by the total dose of FSH administered.

OSI can only be calculated after a first stimulation cycle, but it seems a promising marker capable of expressing the sensitivity of the ovary, without being conditioned by the stimulation protocol.

Huber et al. also studied a large population undergoing IVF and demonstrated that OSI has a normal distribution in the study population [18]. After applying the standard statistical procedures for normal populations, they defined poor, normal, or high responding patients based on the OSI value. The groups showed significant differences in all major outcomes. Furthermore, in accordance with our analysis, it emerged that OSI is a stronger predictor of live birth rates than the number of oocytes retrieved at pick up [18,19,20].

Li and collaborators [19] evaluated the inter-cycle variability of the OSI value and the number of oocytes and confirmed that OSI has a higher intra-class correlation coefficient (ICC) between two stimulation cycles than the number of oocytes.

OSI is a patient-related marker, described as the ovarian sensitivity to exogenous stimuli, is an intrinsic characteristic that has little subject to change. It is therefore a valid marker of ovarian sensitivity that could possibly be introduced in the logarithms used for calculating the dose of gonadotropins to be used, obviously in patients who have already undergone a first stimulation cycle. It should be noted, however, that the main disadvantage of OSI lies in the intrinsic characteristic of being operator-dependent.

### Limitations Section

The main limitations of our study are related to its retrospective and monocentric character and sample size. It was very difficult to recover all the necessary data for the calculation of the FORT FOI and OSI indices; we had to exclude several patients for whom we were unable to recover all data. For further validation of the results, it would be desirable to design a prospective study, with a larger study population and a longer observation period. We also had to exclude from our analysis some patients who had achieved pregnancy but had not yet given birth. Furthermore, a multicentric study would allow us to evaluate whether the use of different stimulation protocols or lower gonadotropin doses can lead to differences in results—in our center, minimum gonadotropin dose is 225UI, the maximum dose is 600 IU. Furthermore, we do not have any information about the ploidy of the embryos, as it was not possible to perform PGTA.

## 5. Conclusions

Our findings suggest that the best index, among those analyzed that predicts cIR and cLBR is OSI. Both OSI and FOI predict embryo culture success but OSI is more accurate. OSI, FOI, and FORT are significantly related to the number of MII oocytes obtained. Only OSI is correlated with fertilization rate.

## Figures and Tables

**Table 1:** Characteristics of the study population. Culture: group of patients who obtained a successful embryo culture. No culture: group of patients who did not obtain a successful embryo culture. N and percentage for categorical variables and median and median + (25th percentile–75th percentile).

	Culture		No Culture		*p* Value
	N = 298	69.46%	N = 131	30.54%	
IVF technique used					0.426
FIV	170	57.1%	64	48.9%	
FIV trasformed into ICSI	5	1.7%	2	1.5%	
ICSI	119	39.9%	62	47.3%	
ICSI (freeze)	4	1.3%	3	2.3%	
Male smoking					0.125
Never	186	65.0%	70	56.9%	
Yes	57	19.9%	36	29.3%	
Former smoker	43	15.1%	17	13.8%	
Female smoking					0.835
Never	232	79.4%	100	76.9%	
Yes	33	11.3%	16	12.3%	
Former smoker	27	9.3%	14	10.8%	
Type of infertility					0.545
Primary	175	58.7%	81	61.8%	
Secondary	123	41.3%	50	38.2%	
Age, years	39.9	[39.0–41.3]	40.58	[39.4–41.7]	0.010
BMI, kg/m^2^	22.9	[20.9–26.3]	23.05	[20.5–25.6]	0.206
FSH, IU/l	6.7	[5.0–8.0]	7.6	[6.3–9.9]	<0.001
AFC	14.0	[10.0–19.0]	10.0	[6.0–14.0]	<0.001
Oocytes retrieved	10.0	[7.0–16.0]	5.0	[2.0–8.0]	<0.001
AMH, ng/mL	1.75	[1.0–3.0]	1.0	[0.6–1.9]	<0.001
Gn total dose, UI	5400	[4050–6772]	6600	[5400–7350]	<0.001
Duration of Gn treatment, days	11.0	[10.0–12.0]	12.0	[10.0–13.0]	0.018
